# Bigger doesn’t mean bolder: behavioral variation of four wild rodent species to novelty and predation risk following a fast-slow continuum

**DOI:** 10.1186/s12983-020-00376-8

**Published:** 2020-09-21

**Authors:** Ian Nicholas Best, Pei-Jen Lee Shaner, Hsuan-Yi Lo, Kurtis Jai-Chyi Pei, Chi-Chien Kuo

**Affiliations:** 1 Biodiversity Program, Taiwan International Graduate Program, Academia Sinica, Taipei, Taiwan; 2 Department of Life Sciences, National Taiwan Normal University, Taipei, Taiwan; 3 Taipei Zoo, Taipei, Taiwan; 4 Institute of Wildlife Conservation, College of Veterinary Medicine, National Pingtung University of Science and Technology, Neipu, Pingtung Taiwan

**Keywords:** Predation risk, Behavior, Rodent, Foraging, Novelty, Leopard cat, Defensive, Fast-slow continuum

## Abstract

**Background:**

Understanding how wild species respond to novel situations with associated risk can provide valuable insights for inter-specific behavioral variation and associations with pace-of-life (POL). Rodents, a globally distributed and diverse taxonomic group, have been the subjects of countless studies emulating risky situations. Controlled laboratory experiments with a focus on wild-caught species provide the opportunity to test fine-scale behavioral responses to contexts of risk with ecological implications. For example, assessing the importance of predator cues eliciting antipredator responses, as well as whether wild rodents embody behavioral plasticity and repertoires, illustrated by habituation and variation in behavioral traits, respectively.

**Results:**

In this comparative study, we examined multiple behavioral responses of four rodent species in eastern Taiwan (three native species *Mus caroli*, *Apodemus agrarius, Rattus losea*, and one invasive, *Rattus exulans*) exposed to an unfamiliar microenvironment and novel cue from an allopatric predator, the leopard cat (*Prionailurus bengalensis*). All wild-caught animals were subjected to two consecutive nights of experimental trials in a laboratory setting. Behavioral responses to a novel situation during the first trial differed between species; smaller species investing more time in non-defensive behaviors compared to the larger species. More specifically, the smaller species *M. caroli* and *A. agrarius* allocated more time to exploration and foraging, whereas the larger rat species *R. exulans* and *R. losea* spent more time motionless or concealing. During the second trial, the addition of leopard cat cues did not elicit antipredator behaviors, but rather, rodents were found to exhibit increased non-defensive behaviors, specifically foraging efforts.

**Conclusions:**

Our results suggest that these four species do largely follow a behavioral fast-slow continuum with the two smaller mice species demonstrating increased boldness in a novel context compared to the larger rat species. Also, the wild populations of rodents in eastern Taiwan may be naïve to leopard cats. Finally, the rodents in our study demonstrated habituation to the microenvironment, indicating they possess adaptive capacity.

## Background

The pace-of-life (hereafter POL) hypothesis suggests that closely related species should differ in a suite of physiological (e.g. metabolic rate) and morphological (e.g. body size) traits that have coevolved with their respective life-histories in a fast-slow continuum [1–3]. It is well documented in mammals that small species embodying fast-paced life histories tend to favor current reproduction over survival since they are shorter lived, as opposed to larger slow-paced strategists with longer lifespans prioritizing survival over reproduction – thereby exemplifying the fast-slow continuum [1, 4]. A growing body of research has postulated that behavior is linked to POL [5–9]. More specifically, species with fast life histories may also be more likely to express behaviors for increased boldness, fast exploration and foraging for resources, even at risk to their survival [3, 10, 11]. Additionally, species with a slower POL are predicted to exercise more caution in response to risk [12, 13]. Therefore, a comparative study investigating the behavioral responses of several species to risky contexts could provide a means of testing the links between POL and behavior.

An increasing number of studies have been devoted to animal personality, which can be defined as between-individual variation in behaviors that are consistent over time and across contexts [5, 6, 14]. Among the personality trait axes defined [6], shyness-boldness, exploration-avoidance, and activity are commonly applied to risk-related studies [15–18]. Behavioral types, or personality traits, have direct implications for fitness, since they can govern habitat use, social interactions, dispersal and responses to risk [15, 19]. Given that many species have been found to exhibit intra-individual consistencies and inter-individual variation for behaviors and habituation [15, 17, 20], this is an important aspect to consider in a comparative study examining behavioral responses of multiple species to novelty.

Sexual variation in life history traits and subsequent dissimilarities in risk-taking behavior may also be expected for many species, particularly those with polygamous mating and/or female parental care [21, 22]. Therefore, in mating systems where the reproductive success of males may be more variable than females, the former sex may demonstrate more boldness, be more proactive in exploration of novel situations and more likely to take risks to acquire resources [23, 24].

Studies manipulating predation risk have been executed in both laboratory and field settings and often use olfactory cues of predators [25–28]. Laboratory studies provide the ability to control for extraneous or unwanted factors, as well as the simulation of ‘micro-environments’ and observation of behaviors at a very fine scale. Risk-related laboratory experiments often involve rodents tested in a maze or open-arena and exposed to a stimulus, e.g. novel object, predator odor [28–30]. Many studies performed on captive-reared rodents have found positive effects of predator odors; acting as deterrents [26, 30]. Other studies that have tested wild-caught rodents have found no effect of predator odor [17, 26, 27, 31, 32]. Wild rodents may not have the same behavioral responses as captive ones, especially since they have higher genetic variation promoting greater diversity of morphological and behavioral traits [33, 34]. Additional explanations for the inconsistencies in prey responses to predator odors include species traits, individual personality and differences, physiological state, and fear conditioning and habituation [17, 28].

Rodents are common prey to a suite of predators, and in order to keep pace in an evolutionary arms race they have been equipped with antipredator responses [35, 36]. Predator cues, such as odors, elevate risk for prey and can instigate defensive behaviors including increased vigilance, avoidance of areas, hiding, immobility and decreased activity [27, 37, 38]. These antipredator responses come at a cost, for example, according to the ‘predator sensitive foraging hypothesis’ the risk of predators will constrain prey foraging activity and efficiency due to an increase in defensive behaviors, such as vigilance and motionlessness [36, 39, 40].

In Taiwan, a mammalian predator of rodents is the leopard cat (*Prionailurus bengalensis*) (Kerr 1972). Leopard cats, the sole-remaining native cat, are classified as endangered and protected under Taiwan’s Wildlife Conservation Act. This wild felid currently occupies a fraction of its once island-wide distribution [41], which is limited to a few regions in the western part of Taiwan where the sympatric rodents are a major constituent of the cat’s diet [42]. The same murine rodent species are also distributed in eastern Taiwan, where leopard cats are no longer found. These rodents, which include both native and exotic species, vary in body size and associated life history traits [43, 44]; the larger rat species can be more than ten times the size of the smaller mice species. Additionally, the smaller mice species have shorter life spans and reach sexual maturity at an earlier age compared to the larger murid rats, as well as differences in fecundity and number of reproductive periods [4, 45, 46]. Therefore, despite occupying similar habitat, the various murid species in eastern Taiwan embody variation in their POL following a fast to slow continuum [4, 6], and may exhibit different strategies regarding exploration, acquisition of resources, and assessing risk. Furthermore, these differences in strategies may also be reflected in their behaviors; smaller species demonstrating more boldness in response to risky situations compared to the larger species [6, 11].

The Pacific rat (*Rattus exulans*) is one invasive species that has been expanding its range and invasion front in eastern Taiwan (I. Best, unpublished data). Invasive species that are widely distributed are generally thought to be generalists and ecologically plastic [47]; in order to invade a diverse array of habitats. Furthermore, it has been posited that generalist species are more likely to confer boldness and less likely to express neophobia compared to specialist counterparts [8, 48]. Therefore, an invasive rat may respond differently than native species when exposed to a novel situation.

**Fig. 1:**
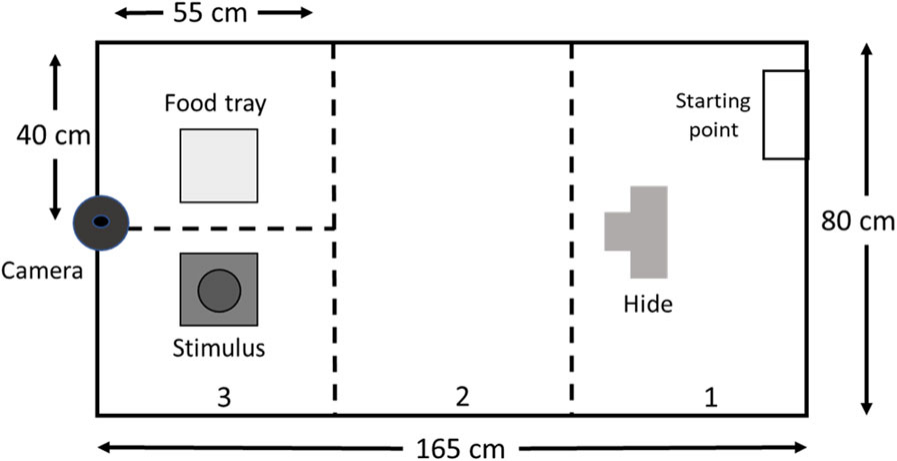
Schematic of the base of the test arena in the trials. The divisions of the different sections are marked with dashed black lines. The numbers correspond to the specified section. Section 3 included both the area with the food tray and stimulus. Stimulus refers to treatment apparatus

The present-day distribution of leopard cats and rodents allowed us to test the effects of cues from an allopatric predator on multiple rodent species. Since none of the rodents included in our study had ever encountered leopard cats, we could provide a first-step approach to evaluate whether these antipredator behavioral responses are conserved or lost rendering the rodents naïve. In this study, we conducted a laboratory experiment (Fig. 1) on four wild rodent species in eastern Taiwan and measured their behavioral responses (Refer to Table 1 for a description of each behavior) to a novel environment and a novel predator cue (leopard cat odor). Our experimental design also enabled us to investigate whether the rodents would habituate to novel conditions. Therefore, our objectives were to examine whether (1) there would be inter-sexual behavioral differences to the novel environment, (2) there would be inter-specific variation in behavioral responses to the novel environment and leopard cat odor, following a fast-slow continuum, (3) leopard cat odor would elicit defensive behaviors, and (4) rodents would become habituated to the experimental trials.

**Table 1:** Behavioral response variables measured from experimental trials

Response	Unit measured	Definition
*Exploring*^*a*^	Time (s.)	Locomotive activity, investigation of the arena and objects, climbing
*Foraging*^*a*^	Time (s.)	The subject consumed and/ or searched for seeds in the foraging tray
*Motionless*^*b*^	Time (s.)	The subject remained stationary (except for breathing); body remained stationary with occasional head scanning
*Concealing*^*b*^	Time (s.)	The subject was in the hide (at least 75% of the body was concealed); the head was slightly exposed from the hide
*Grooming*	Time (s.)	The subject was grooming, e.g. licking or rubbing
*Consumption*^*c,*d^	Weight (g)	The amount of seeds consumed (± 0.1 g). Calculated by subtracting the remaining amount from the initial 5.0 g
*Foraging events*^d^	No. occurrences	The number of events of foraging of the test subject
*Latency to forage*^d^	Time (s.)	The amount of time before the subject started foraging
*Jumping*^d^	No. occurrences	The subject actively jumped; all four paws left the base of the arena
*Contact*	No. occurrences	The subject investigated or had tactile contact with the treatment apparatus, e.g. sniffing or biting

Notes: seconds (s.), number of (no.) occurrences, grams (g). Response variables adapted from [29, 49, 50]. ^a^ refers to behaviors included in the ‘Non-defensive’ behavioral category. ^b^ refers to behaviors included in the ‘Defensive’ behavioral category. ^c^ this behavior was transformed to *Consumption ratio*, by calculating the food eaten proportional to body weight and expressed as a percentage. ^d^ indicates behaviors removed from the focus of the main text, see Supplementary material (Table S4, Fig. S1, Figure S2) for results. For a complete list of the means and standard errors of all the response variables, please refer to Supplementary material (Table S1 and S2)

## Results

### Behavioral responses to a novel environment

**Fig. 2:**
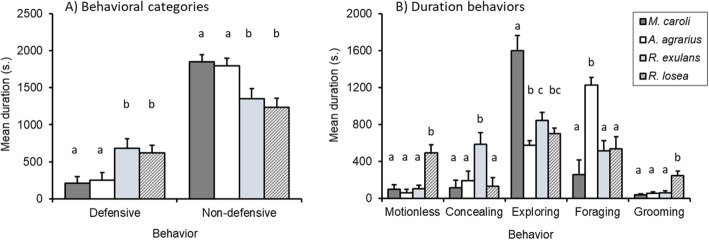
Mean duration (out of 2100 s) of behavioral responses between species during the first trial; **a** Defensive and non-defensive categories, **b** All duration-based behaviors. Error bars represent standard error of the mean. Differences in letters above each response variable indicate significant differences based on post hoc analysis between species groups

Our analysis found significant effects of species for all behavioral responses (Table 2, Fig. 2). The two mice species, *M. caroli* and *A. agrarius*, spent more time performing non-defensive behaviors, whereas the two rat species, *R. exulans* and *R. losea*, invested more time in defensive behaviors during the first trial (Fig. 2a). Furthermore, during the first trial the species *M. caroli* spent the most time exploring, while *A. agrarius* spent the most time foraging (Fig. 2b). *R. losea* spent more time motionless and grooming compared to the other species, and *R. exulans* spent the most time concealing (Fig. 2b).

**Table 2:** Behavioral responses of the first trial for the factor species, sex and their interaction. Significant values are displayed in bold

Response	Factor	Wald χ^2^	*df*	*P*
*Defensive*	Species	19.03	3	**<0.001**
	Sex	1.90	1	0.168
	Species × Sex	4.90	3	0.179
*Non-defensive*	Species	22.07	3	**<0.001**
	Sex	4.86	1	**<0.05**
	Species × Sex	11.80	3	**<0.01**
*Exploring*	Species	38.90	3	**<0.001**
	Sex	0.62	1	0.433
	Species × Sex	2.26	3	0.521
*Foraging*	Species	38.55	3	**<0.001**
	Sex	24.68	1	**<0.001**
	Species × Sex	30.55	3	**<0.001**
*Motionless*	Species	48.77	3	**<0.001**
	Sex	0.94	1	0.332
	Species × Sex	27.92	3	**<0.001**
*Concealing*	Species	57.99	3	**<0.001**
	Sex	29.28	1	**<0.001**
	Species × Sex	41.71	3	**<0.001**
*Grooming*	Species	32.14	3	**<0.001**
	Sex	3.50	1	0.061
	Species × Sex	6.86	3	0.076

Sex had significant effects for the responses *foraging*, *concealing* and the *non-defensive* behavioral category; with significant interactions of sex and species for *foraging*, *motionless*, *concealing*, and *non-defensive* (Table 1). The males of all species combined spent more time exhibiting non-defensive behaviors, specifically foraging, whereas females spent more time concealing. At the species level, males of *R. losea* invested significantly more time foraging compared to females (both *P* < 0.005). Additionally, females of *R. losea* spent more time concealing in the hide (*P* < 0.05). Despite the absence of significant differences, we observed a trend of the males of each species group allocating slightly more time to foraging; as well as the females of *M. caroli* and *A. agrarius* staying concealed for marginally longer durations (Table S1). Females of the species *R. exulans* spent more time motionless compared to males (*P* < 0.05).

### Behavioral responses to leopard cat odor

**Fig. 3:**
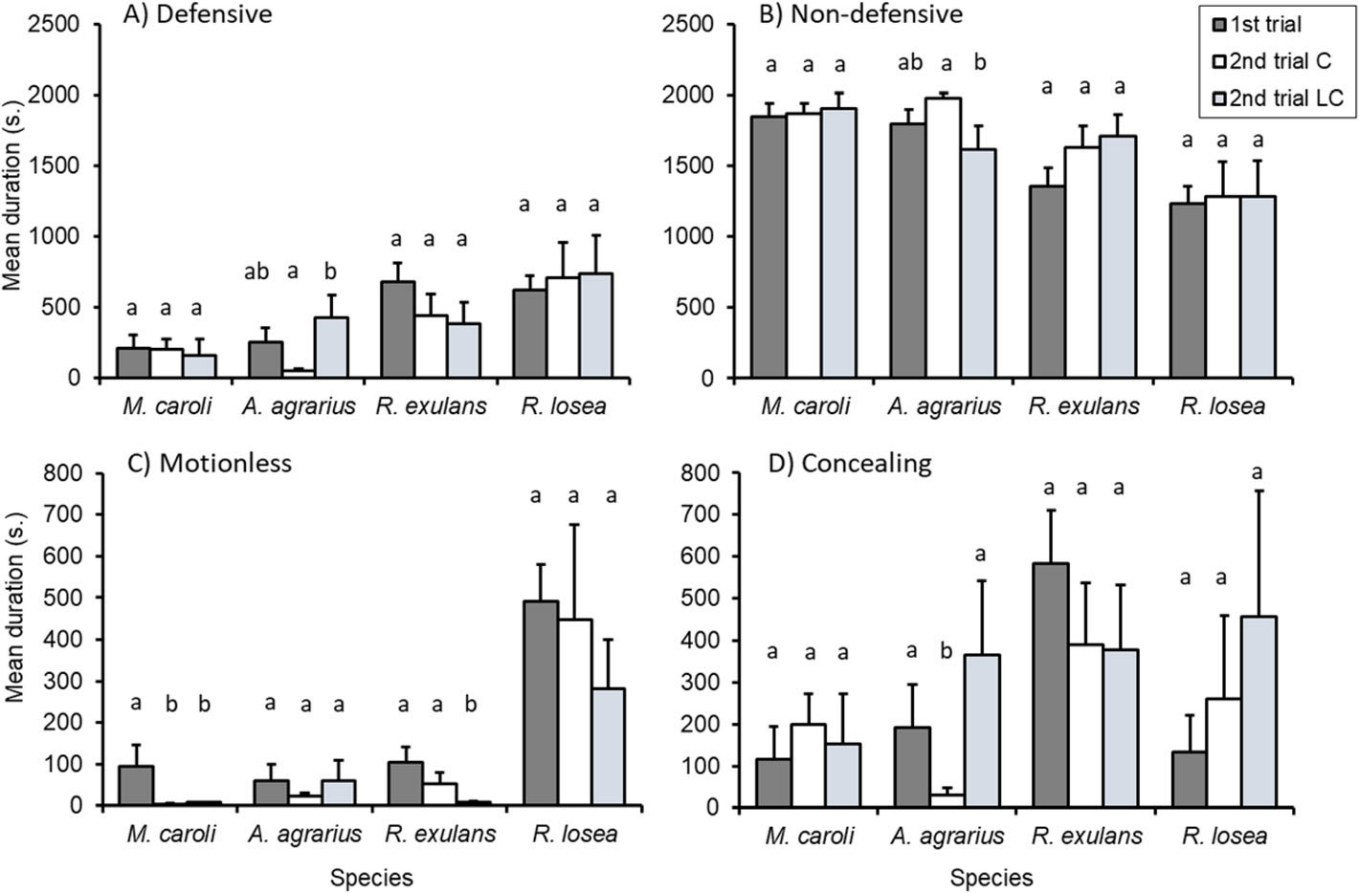
Mean duration (out of 2100 s) of **a** Defensive and **b** Non-defensive behavioral categories, **c** Motionless, and **d** Concealing. Each response variable is compared between species, treatment group and trial. 1st trial comprises both treatment groups. ‘2nd trial C’ refers to the control group during the second trial. ‘2nd trial LC’ refers to the leopard cat odor group during the second trial. Error bars represent standard error of the mean. Differences in letters above each species indicates significant differences based on post hoc analysis between subgroups: 1st trial, 2nd trial C, 2nd trial LC

We found leopard cat odor to have no significant effect on the *non-defensive* behavioral category (Table 3). There was a significant interaction between species and treatment for the *defensive* behavioral category, but not *non-defensive* (Table 3). More specifically, the leopard cat odor treatment group of the species *A. agrarius* spent more time exhibiting defensive behaviors compared to the control group (Fig. 3a).

**Table 3:** Behavioral responses for the effects and interactions of trial, treatment and species. Significant values are displayed in bold

Response	Factor	Wald χ^2^	*df*	*P*
*Defensive*	Trial	0.06	1	0.800
	Treatment	1.99	1	0.159
	Species	51.57	3	**<0.001**
	Trial × Treatment	0.06	1	0.810
	Trial × Species	4.63	3	0.201
	Treatment × Species	35.70	3	**<0.001**
*Non-defensive*	Trial	2.46	1	0.117
	Treatment	0.27	1	0.603
	Species	22.67	3	**<0.001**
	Trial × Treatment	0.01	1	0.979
	Trial × Species	3.23	3	0.358
	Treatment × Species	6.15	3	0.104
*Exploring*	Trial	12.18	1	**<0.001**
	Treatment	0.39	1	0.531
	Species	155.08	3	**<0.001**
	Trial × Treatment	0.02	1	0.882
	Trial × Species	24.71	3	**<0.001**
	Treatment × Species	1.63	3	0.653
*Foraging*	Trial	4.96	1	**<0.05**
	Treatment	0.10	1	0.756
	Species	42.90	3	**<0.001**
	Trial × Treatment	1.27	1	0.261
	Trial × Species	11.86	3	**<0.01**
	Treatment × Species	4.05	3	0.257
*Motionless*	Trial	26.07	1	**<0.001**
	Treatment	0.18	1	0.668
	Species	93.87	3	**<0.001**
	Trial × Treatment	0.16	1	0.691
	Trial × Species	19.06	3	**<0.001**
	Treatment × Species	17.17	3	**<0.005**
*Concealing*	Trial	3.06	1	0.080
	Treatment	4.14	1	**<0.05**
	Species	31.80	3	**<0.001**
	Trial × Treatment	0.02	1	0.882
	Trial × Species	7.01	3	0.071
	Treatment × Species	26.26	3	**<0.001**
*Grooming*	Trial	10.73	1	**<0.005**
	Treatment	1.29	1	0.256
	Species	51.16	3	**<0.001**
	Trial × Treatment	0.23	1	0.633
	Trial × Species	11.45	3	**<0.05**
	Treatment × Species	4.71	3	0.194

For both behavioral categories there were significant differences between species (Table 3). The two mice species *M. caroli* and *A. agrarius* spent less time exhibiting defensive behaviors and more time performing non-defensive behaviors compared to the larger rat species, *R. exulans* and *R. losea* (Defensive: *M. caroli* compared to *R. exulans* and *R. losea*, both *P* < 0.005; *A. agrarius* compared to *R. exulans* and *R. losea*, both *P* < 0.001; Non-defensive: *M. caroli* compared to *R. exulans* and *R. losea*, both *P* < 0.005; *A. agrarius* compared *R. exulans* and *R. losea*, both *P* < 0.05).

**Fig. 4:**
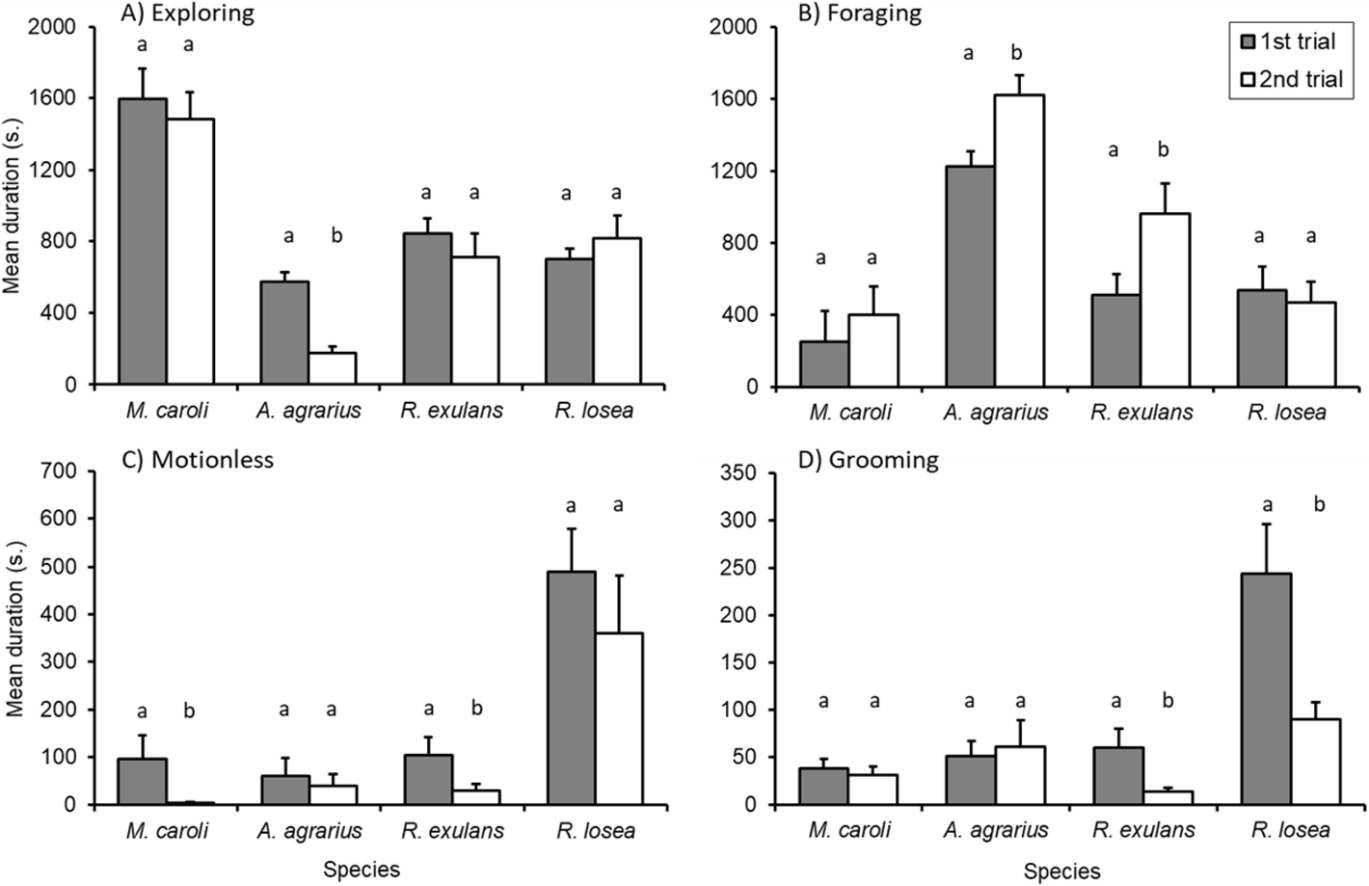
Mean duration (out of 2100 s) of behaviors **a** Exploring, **b** Foraging, **c** Motionless, **d** Grooming. Each response variable is compared between species and trial. Error bars represent standard error of the mean. Differences in letters above each species indicates significant differences based on post hoc analysis between 1st trial and 2nd trial for that species

Leopard cat odor had significant effects on *concealing for A. agrarius* and *motionless* for *R. exulans*. *A. agrarius* exposed to the predator odor spent more time concealing than their counterparts without exposure (Fig. 4d). These results may be explained by within-individual consistency in concealing behavior across the two trials for two individuals of *A. agrarius* (1 male, 1 female) that were included in the leopard cat odor treatment group (Table S5, S6). These two individuals were also outside the upper 95% confidence interval for the mean of time spent concealing. The control group of the species *R. exulans* spent more time motionless compared to the group exposed to leopard cat odor (Fig. 4c). These results may not be so much of an effect of the leopard cat odor, but rather between-individual variation for the behavior (Table S5, S6).

Leopard cat odor did not discourage rodents from making contact with the treatment apparatus (Wald χ^2^ = 0.04, *P* = 0.85), nor was there an interaction between treatment type and species for this behavior (Wald χ^2^ = 0.55, *P* = 0.91). There were significant differences between species for the behavior *contact* (Wald χ^2^ = 7.98, *P* < 0.05; Figure S2). *M. caroli* had the highest number of contacts, irrespective of treatment type (*M. caroli* compared to *A. agrarius*, *R. exulans* and *R. losea*, all *P* < 0.05).

### Behavioral responses across trials

The amount of time each species group exhibited *defensive* or *non-defensive* behaviors did not differ significantly between trials (Table 3). However, trial had significant and species-specific effects on the behaviors *exploring*, *foraging*, *motionless* and *grooming* (Table 3).

With the results of both treatment groups (control and leopard cat odor) in the second trial combined, *A. agrarius* displayed a decrease in time spent exploring on the second night (Fig. 4a), and both *A. agrarius* and *R. exulans* significantly increased the time spent foraging (Fig. 4b). Also, during the second trial the species *R. exulans* and *M. caroli* reduced the amount of time spent motionless (Fig. 4c), and *R. exulans* and *R. losea* spent significantly less time grooming (Fig. 4d).

### Intra-individual consistency of behaviors

Based on our analysis of repeatability, rodent ID had significant effects for all behaviors except *grooming* for the first conditional model (without fixed effects; Table S5); and significant effects for all behaviors except *exploring* and *grooming* for the second conditional model (with fixed effects; Table S6). These results suggest that for most behaviors there was within-individual consistency (repeatability) and between-individual variation (Table S5 and S6).

## Discussion

In the present study, inter-specific variation was observed for behavioral responses in an experiment testing novelty. On average, the smallest species in our sample, *M. caroli*, spent the most time exhibiting non-defensive behaviors, predominantly comprised of exploring. The other mouse species and second smallest, *A. agrarius*, spent the most time foraging. Contrarily, the two rat species in our experiments spent considerably more time demonstrating defensive behaviors; *R. exulans* spent the most time hiding and *R. losea* was motionless for the longest cumulative period. *R. losea* also spent the most time grooming during the first trial, which could reflect nervousness or be a reaction to a stressor [15, 51], such as the novel environment. In addition to being larger, the rat species, included in the present study, have longer lifespans and reach sexual maturity later compared to the two mice species [46]. Our results suggest that the different species fit a fast-slow continuum with predicted associated behaviors [6–9], demonstrated in the present study by the smaller mice species performing a higher proportion of riskier behaviors (exploration, activity and foraging) in a novel situation, in contrast to the larger rat species. The smaller species in our study, *M. caroli* and *A. agrarius*, prioritized fast exploration or acquisition of resources, as oppose to defensive behaviors. *A. agrarius* favored the acquisition of resources over exploration or cautious behavior in an unfamiliar situation. These results may indicate a trade-off favoring current fitness returns compared to future expectations [10, 12]. Similarly, Vasquez [52] studying foraging behavior of three different Chilean rodent species varying in body size found that under increased risk the largest species was the most cautious.

The response variables foraging and concealing were found to differ significantly between sexes. At the rodent community level (all species combined), males spent more time foraging compared to females for their first trial. Since male rodents generally have less parental investment and are promiscuous, it can be expected that there will be sex-related differences for trade-offs between risk and reward [21, 36]. Therefore, males may have a higher propensity to expose themselves to risk in return for a reward [22]; in the case of the present study, risk of exposure in an unfamiliar environment and a reward of access to food. Male rodents are generally larger than females [36, 53], therefore, they may also have a higher food intake potential [54], as was observed in the present study. Overall, the defensive behavior concealing was higher for females, which indicates that in response to a novel, unfamiliar situation females exercised more caution. Adult, sexually mature females may show a preference for defensive behaviors in a novel context since the risks may outweigh rewards as they incur higher costs for parental care [36]. Our results are consistent with other studies examining behavioral responses to high risk situations [22, 25], with female rodents exhibiting a higher proportion of defensive behaviors, such as hiding, compared to males.

The presence of leopard cat cues during the second trial did not have effects on the defensive and non-defensive behavioral categories. There was, however, species-specific significant effects (increased concealing in *A. agrarius* and decreased motionlessness in *R. exulans* in response to leopard cat odor), which can most likely be attributed to within-individual consistency and between-individual variation. Individuals of *A. agrarius* that were assigned to the leopard cat odor treatment group also were more inclined to hide, which was observed consistently over both trials. Between-individual variation could explain how individuals of *R. exulans* in the control group spent more time motionless during the second trial compared to those in the predator odor group. Repeatability of behaviors over time and even across varying situations has been observed in similar experiments [15, 55, 56], and can even outweigh the effects of predator odors [17].

The predator odor failing to elicit aversive behaviors in the present study conforms with many other studies that have exposed rodent species from wild populations to predator cues both in lab [17, 25, 57] and field [32, 58, 59] contexts. Furthermore, many studies that have found significant effects of predator odors performed their experiments on captive-bred rodents [22, 26, 49, 50]. The domestication process of captive rodents may lead to an inhibition of behavioral variation and adaptability [33, 60], resulting in more pronounced responses to foreign odorous stimuli. There is a growing consensus stipulating that for wild prey populations predator odors alone may not evoke strong antipredator responses [6, 32, 61], but in turn, a combination of factors, including physiology, type of perceived risk, and habituation [28]. Indirect risk factors, such as illumination and vegetation cover, have been found to play larger roles in governing rodent foraging behavior compared to direct predator cues, such as odor [61, 62].

Since leopard cats have been absent in eastern Taiwan for several decades, and therefore numerous generations of the local rodent species, it is possible these respective rodents have lost the ability to discriminate the odor. Additionally, other small carnivores in eastern Taiwan that are capable of predating on rodents, such as the lesser civet (*Viverricula indica*) and feral cats (*Felis catus*), occur at low densities (I. Best, unpublished data) or rodents are not a main prey item for them [63, 64]. Antipredator responses are very costly [65] and if a given trait no longer serves a purpose it is likely that it will be selected against and lost [66]. Furthermore, according to the naiveté hypothesis prey are not expected to discriminate and respond accordingly to novel predators due to no previous encounters [67]. In Australia, the invasion of cane toads (*Rhinella marina*) prompted the relocation of native Northern quolls (*Dasyurus hallucatus*) to predator-free islands, and they have lived in these conditions for multiple generations [57]. Jolly et al. [57] compared responses of quolls from both the predator-free island population and mainland Australia to native predator cues. Opposite to the mainland quolls, the island population showed no aversion to the predator odors. For the current study, despite the possibility that rodents inhabiting leopard cat-free regions are naïve to the predator and are unable to recognize their odors, further research testing rodents in areas where leopard cats are present is necessary to affirm this prediction.

Given the lack of predator odor effects on rodent behavior, we were able to examine behavioral responses across trials. Two out of the four species showed significant increases in amount of time foraging, and *M. caroli* did spend more time foraging despite the difference not being significant. *M. caroli* and *R. exulans* also significantly decreased the amount of time spent motionless during the second trial. Therefore, the three species, *M. caroli, A. agrarius* and *R. exulans*, demonstrated a trade-off in defensive behaviors, as well as exploration, for access to food resources, which can be indicative of boldness [6, 16, 18]. Even though exploring can be constituted as a non-defensive behavior conferring some boldness [5], individuals are still able to keep some level of vigilance [6, 10] whereas with foraging, vigilance is sacrificed to a much higher degree [68].

Our results provide further support that wild populations of rodent species can have behavioral plasticity, as habituation can be linked to phenotypic plasticity [15, 69]. In the case of the present study, the increase in foraging activities and exploitation of the food patch can reflect learning and be a measure for information processing [70]. Moreover, after accumulating sufficient knowledge of an initially unfamiliar environment (through repeated exposure), an optimal strategy could be to switch from exploration to exploitation of resources for an energetic reward [71, 72]. Additionally, the variation between individuals that was observed in our experiments (Table S5 and S6) could also indicate large behavioral repertoires of the wild populations [73]. Therefore, the behavioral differences did not stop at the species level, but also within species at the individual level. A broad behavioral repertoire could also have implications for fitness under a changing environment – increased human activity and disturbance. A species with a wider behavioral range (boldness-shyness) may be more resilient to disturbances [8, 74].

We find it unlikely that the increases in foraging activity observed were stress-induced or a product of our experimental procedure. Animals were food deprived for the same amount of time on both days of testing and were provided with ample food upon return to their housing cage after completing the first trial. Moreover, high levels of acute stress on rodents may inhibit food intake and prompt defensive behaviors [75, 76]. We also consider it unlikely that the addition of novel objects (treatment apparatus) during the second trial masked the effects of the predator odor by instigating strong neophobic responses, since a majority of the test animals displayed the opposite response indicated by a decrease in defensive behaviors and exploration, irrespective of treatment type.

Although our results demonstrate changes in behavior across trial and context, likely reflecting habituation, we acknowledge that the short inter-trial interval may have influenced this result. Since a main objective of this study was to test the immediate responses of wild-caught rodents to a novel environment and predator odor, our experimental design did not incorporate lengthy intervals between testing. Additionally, many predator odor studies have used similar experimental durations and intervals [17, 30, 49, 50]. Our study does provide a first-step approach for evaluating inter-specific habituation to a microenvironment in a controlled setting for the included species. To further substantiate our results, future studies could adopt a longer period between testing and incorporate repeated measures that more appropriately fit the research questions.

The largest species included in our study, *R. losea*, had contrasting responses compared to the other species during the second trial. Namely, the species failed to exhibit significant increases in any of the non-defensive behaviors. The species did decrease the amount of time grooming, however, possibly suggesting a decrease in reactionary stress [51]. The former results may indicate different rates of habituation between the species. On average, *R. losea* ranged from three to ten times larger in size than the other species. With the predictions of the POL following a behavioral fast-slow continuum [6, 8, 12], *R. losea* would be expected to be the most cautious species in our study, therefore, it could also be possible that this species would habituate to novelty at a slower pace. Larger species with slower life history traits tend to be more cautious with stronger neophobia responses [6, 8, 10], therefore habituation to a novel situation with associated risk maybe slower compared to smaller species.

The invasive species, *R. exulans*, somewhat surprisingly spent the most time concealing during the first trial. However, the second trial for this species comprised a drastic reduction staying motionless with an increase in foraging. The average amount of time spent hiding was also lower, though the difference between the two trials was not significant. These results demonstrate the plasticity and habituation potential of the rat, which may be characteristic of an invasive species [5, 77, 78]. Additionally, the initial caution the species exercised could also be somewhat indicative of their strategy for occupying novel environments – not overly bold to a degree of recklessness. The species could benefit from processing information and assessing risk about the new environment from a safe refuge in addition to exploration [79, 80]. To better understand the habituation potential and rate of invasive species, further studies adopting a comparative approach involving multiple invasive and native species will be necessary.

Interestingly, in tandem with the range expansion of *R. exulans* in eastern Taiwan, *A. agrarius* has been experiencing population declines [81]; I. Best, unpublished data]. In the present study, we observed *A. agrarius* to be the most voracious foragers exposing themselves to risk for the longest periods of time. The lack of defensive behaviors to the simulated cues of risk in our experiments (novel environment and objects) may suggest that they have an increased vulnerability to predators, biological enemies and other disturbances in the wild.

Our assessment testing intra-individual consistency found most of our measured behaviors to be repeatable, supporting between-individual variation and likely behavioral types. Individuals in our trials fit a spectrum of boldness and exploration/ activity. Some caution should be exercised in the interpretation and application of these results due to the experimental design of our study. An initial aim of ours was to test inter-specific behavioral responses to a predator cue, therefore, the necessary addition of the treatment apparatus during the second trial changed a context parameter, which may impede validity and statistical power of repeatability tests [82]. Despite this limitation, this comparative, exploratory study does provide a foundation for inter-individual variation and within-individual repeatability of behaviors on a multi-species level. We suggest that future studies employ the appropriate methodology and design better suited to examine personality traits of individuals amongst different species to advance understanding of behavioral plasticity in ecological contexts.

## Conclusions

Most studies to date examining behavioral and life history covariation have largely focused on individuals or populations of a single species [6, 9, 83], therefore our pioneer study provides further insight for the association between behavior and POL, exemplified by inter-specific behavioral variation in accordance with a fast-slow continuum. In a novel microenvironment, the smaller, “faster” species of mice displayed more proactive behaviors conferring boldness, whereas the larger, “slower” rat species exercised more caution. Our findings also suggest that these four species of rodents in eastern Taiwan may be naïve to leopard cat cues, indicating that antipredator behaviors may be learned from experience. However, further research is required to uncover this assumption. Finally, despite the addition of a predator odor and novel object, we observed a trend for an increase in non-defensive behaviors across all species – representing habituation and behavioral plasticity. In the context of regions undergoing landscape changes facilitated by increased human activity and development, as is the case in Hualien County in eastern Taiwan, the survival and success of wild rodents may be dependent on broad behavioral repertoires.

## Methods

### Study area

We conducted our study in Hualien County located in eastern Taiwan. Our experiments took place at National Dong Hwa University, Shoufeng Township, Hualien County (23.90 °N, 121.54 °E). In low-elevation areas of Hualien a variety of habitats supports rodents including the Ryukyu mouse (*Mus caroli*), striped field mouse (*Apodemus agrarius*), lesser ricefield rat (*Rattus losea*), greater bandicoot rat (*Bandicota indica*), as well as the introduced species the house mouse (*Mus musculus*) and Pacific rat (*Rattus exulans*). Leopard cats have been absent in Hualien for multiple decades, but there are historical records of their occurrence in the region [41]. This allowed us to test leopard cat odors as a novel predator cue and to assess whether the native rodents are naïve to leopard cat odors and subsequently lack anti-predator behavioral responses.

### Animal collection and maintenance

Animals were live-trapped using a combination of Sherman (26.5 X 10 X 8.5 cm) and mesh (27 X 16 X 13 cm) traps. We deployed the traps at sites in fields of the agricultural areas of northern Hualien County. Since an objective of this study was to include individuals from multiple rodent species, we sampled different habitat types. All sites were a minimum distance of 500 m apart and only sampled once to ensure that we did not trap the same individual more than once. Wang & Wang [84] reported that large rodent species, such as *R. losea*, rarely move more than 500 m. Traps were baited with sweet potato covered in peanut butter and set in the late afternoon and rechecked first thing the next morning.

Our target species included two mice species, *M. caroli* and *A. agrarius*, and two rat species, *R. exulans* and *R. losea*. The inclusion of these species was due to higher trapping success and for inter-specific representation of rodent communities exhibiting variation in morphological and life history traits. Additionally, since *R. exulans* is an invasive species [81], we wanted to determine if there were any associated behavioral differences from the other native species. Only adults of each species group were included in our experimental trials. Upon capture, target species that met our criteria were examined to determine sex and reproductive status. Reproductive maturity was concluded if testes were descended in the scrotal region for males, and the presence of vaginal perforation and/ or swollen nipples for females. To avoid potential sources of behavioral bias, if females were considered pregnant they were excluded from the trial. We also measured body weight (± 0.1 g), body length (snout to anus, mm) and tail length (anus to tail tip, mm). Animals were kept for a maximum period of 48-h after which they were released at the same site they were captured. We kept rodents in a designated housing room in mesh cages (27 X 16 X 13 cm); with one rodent housed per cage and no more than ten test animals were kept at a given time. Rodents were provided with water and food ad libitum until 10 h before each trial. Additionally, cages contained shredded paper for bedding, a cardboard tube for hiding, and we placed a cover over all cages for additional privacy and to maintain separation. The housing room was maintained at 24 ± 1 °C with natural lighting. Only one researcher entered the housing room to provide water, food and to collect rodents for the trials; this was to minimize disturbance.

### Predator odor

Leopard cat body odor and fecal samples were collected from captive individuals at Taipei Zoo and Pingtung Rescue Center for Endangered Wild Animals. Body odors were obtained by placing clean towels sterilized by an Autoclave in the sleeping areas of that cats’ enclosures for a period of roughly 30 days. This duration was to allow for the towel to be sufficiently permeated with the leopard cat’s odor. Upon receiving the towels, they were cut into smaller segments (15 X 15 cm), which has proven to be an effective size at eliciting antipredator behavioral responses in prey species [30, 85]. The segments were then placed in airtight, re-sealable plastic bags and stored in a − 20 °C freezer until later use. Clean, latex gloves were worn at all times when handling the towels. Fecal samples were also collected from the same donor individuals that provided the body odor samples. Upon request of collection, fresh feces were collected daily, placed in airtight, re-sealable plastic bags and stored in a freezer at − 20 °C. Samples were stored in a freezer for a maximum period of 2 months before use. On the day of experimental trials, fecal samples of the same donor individual were thawed and pooled together. The feces were then crushed and diluted with distilled water to create a mixture with a ratio of 1-part feces (g) and 1-part distilled water (mL). This ratio has been commonly used in other predator odor experiments [26, 86]. We used body odor and fecal samples in concert for our leopard cat odor treatment. Corresponding body odor and fecal samples of the same donor individual were always paired together. We did not consider the combination of both odor types to be an exaggeration of leopard cat cues, since our aim was to simulate high predation risk. Furthermore, predators, such as felids, often leave multiple scent types at areas they visit [87–89].

### Experimental apparatus

Trials were conducted in an open-area test arena (165 cm long X 80 cm wide X 70 cm deep; Fig. 1), which consisted of an opaque, rectangular-shaped box made of plastic material that was non-permeable and easy to clean. The size of the arena was to allow for sufficient exploration and to prevent escape. The arena was divided into three-sections using a non-odorous tape that was clearly visible under low light; in section 1 a PVC tri-entry tube (referred to as the hide; 50 mm diameter) was placed in the center to allow concealing. We thought it was important to include a hide, as evasion and/ or hiding are common defensive behavioral responses of wild rodents when facing risk [36, 90]. In section 3 at the opposite end of arena we placed a foraging tray and the treatment apparatus (present only during the treatment trial) (Fig. 1). The foraging trays (17.3 X 12.1 X 3.8 cm) contained 5.0 g of millet seed mixed thoroughly in 75 g of extra fine sand. Through our preliminary tests and pilot study, we were able to determine millet seed as an appropriate food source. The purpose of including the foraging tray was to assess propensity to forage in a novel environment and risky context, which enabled a metric for boldness to be measured [16, 18, 91].

We affixed a WI-FI enabled surveillance camera (D-Link DCS-936 L; D-Link, Taipei, Taiwan) equipped with infrared capabilities to the upper edge of the interior wall above section 3 (Fig. 1) and positioned the camera to fit the treatment apparatus, foraging tray and hide in the field of view. This camera also provided us with live streaming of all trials. We also used a camcorder (HausBell HDV-302S; USCLOUND Trade Inc., California, USA) with infrared attached to a tripod and positioned to have the interior of the arena in the field of view. The combination of the two cameras ensured there were no blind spots and the whole interior of the arena was fully captured.

The apparatus for the leopard cat odor treatment consisted of a body odor towel segment placed on a tray (22 X 17 X 3 cm) with 5 g of the fecal solution on a petri dish positioned on top. The non-odor control treatment comprised a clean towel segment sprayed with distilled water placed on a tray with an empty petri dish on top. These apparatus are hereafter referred to as treatment apparatus.

### Trial procedures

Our experimental trials took place from September to November 2018 and January to June 2019. All trials were conducted between 18:00 and 23:00, starting after dusk, in a testing room with the lights turned off to reflect natural light conditions and account for the rodents’ active period. Test subjects were food deprived for at least 10 h before each trial since an objective of this study was to examine foraging behavior. All test animals were tested for two consecutive nights. The purpose of the first trial (first night for each animal) was to test the rodents’ responses to a novel environment (test arena), therefore the treatment apparatus was excluded. During the second trial on the successive night, which included the treatment apparatus, the main aim was to assess the rodents’ responses to the predator odor. The order for animals to be tested was randomly selected and kept the same upon the second night to allow for 24 h between each animal’s trials. Test animals were transferred from to the testing room in their cages by the same researcher for each of their trials. The cages were placed in section 1 (Fig. 1) of the test arena and their cage door was left open. Once it was confirmed that the rodent had left their cage and entered the arena, cages were removed, cameras were activated, and the researcher exited the testing room. Trials were able to be viewed from a separate room via a live stream of the surveillance camera, in addition to being video recorded. The duration of all trials was 35 min, which included a 5-min introductory period, followed by a brief disturbance from a researcher (placement of treatment apparatus in the arena), then the remaining 30 min. We selected this length for our trials since our aim was to measure immediate responses to a novel environment and predator cue. Similar trial durations and inter-test intervals have been employed in lab-based predator odor experiments that have tested on rodents [26, 30, 49, 50]. During the first trial, since the treatment apparatus was absent and not placed in the arena, we mimicked the procedure of entering the testing room after the 5-min introductory period to control for any effects on behavior that the disturbance (placement of treatment apparatus) in the second trial would cause. During the second trial, the treatment apparatus was placed in section 3 of the arena adjacent to the foraging tray (Fig. 1). Upon completion of each trial, animals were returned to their cages and housing room. The remaining food content in the foraging trays were sieved and weighed using an electronic scale (± 0.1 g) before replacing the seeds and sand. We thoroughly cleaned the test arena and apparatus using 75% ethanol and allowed at least 30 min for any lingering odors in the testing room to dissipate before starting the next trial.

### Test subjects

Our sample from the experimental trials included 68 test subjects: 13 *M. caroli* (5 male, 8 female; average weight: male = 12. 8 ± 0.7 g, female = 12.6 ± 0.5 g), 16 *A. agrarius* (7 male, 9 female; average weight: male = 27.7 ± 1.8 g, female = 28.2 ± 1.0 g), 22 *R. exulans* (15 male, 7 female; average weight: male = 44.4 ± 1.4 g, female = 33.1 ± 1.6 g), and 17 *R. losea* (10 male, 7 female; average weight: male = 120.4 ± 6.5 g, female = 109.4 ± 5.7 g). We employed a stratified random sampling design to assign a similar number of individuals from each species to either the control group or leopard cat odor group. Each treatment group consisted of 34 rodents (control = 7 *M. caroli*, 8 *A. agrarius*, 11 *R. exulans*, 8 *R. losea*; leopard cat odor = 6, 8, 11, 9, respectively).

### Behavioral response analysis

The videos of all trials were analyzed manually offline and in-depth. The behaviors we scored were *exploring*, *foraging*, *motionless*, *concealing*, *grooming*, *consumption, foraging events, latency to forage, jumping* and *contact* (Table 1).

For each behavior to be considered and scored it would have to last for at least 3 s. We included the ‘head out’ behavior as part of *concealing* because it was not commonly observed amongst the test subjects. Vigilant rearing was also not observed in our trials. Based on our preliminary trials, the test subjects did not display any preference for the different sections of the arena, so the time spent in different sections was not included. We further divided four of the five duration-based behaviors (Table 1) into two categories *defensive* and *non-defensive* defined as exhibiting *motionless* and *concealing*, and *exploring* and *foraging*, respectively. Exploration and foraging are commonly classified as non-defensive behaviors for rodents, whereas motionless and concealing are considered defensive responses to risk [32, 92, 93]. We defined these behavioral categories in order to test our prediction of inter-specific behavioral variation following a fast-slow continuum. *Contact* was only scored during the second trial because the treatment apparatus was absent in the arena for the first trial. *Consumption* was expected to vary across species in our experiment due to the inter-specific size disparity. To account for this, we calculated a *consumption ratio* defined as the amount of food eaten proportional to the animal’s body weight and expressed as a percentage.

We found some of the measured behaviors to be highly correlated; time *exploring* with *jumping*, and time *foraging* with *consumption ratio* and *foraging events* based on Spearman rank correlations (Table S3). Additionally, *latency to forage* was significantly negatively correlated with time *foraging* (Table S3). To avoid redundancy, we excluded the behaviors *consumption ratio*, *foraging events, latency to forage* and *jumping* from the focus and analysis included in the main text. Results of these behaviors are available in the Supplementary material (Table S4). Therefore, time spent *exploring* served as a proxy for exploration and activity [5, 6, 71], and time *foraging* for resource acquisition and boldness [16, 18].

### Statistical analysis

Since our response variables did not meet the assumptions of normality we employed generalized linear models (GLM) and generalized estimating equation (GEE) models encompassing various link functions that best fit the distribution of our data. For longitudinal analysis, GEEs have been found to be very robust, flexible and well-suited models for behavioral data that violate the assumptions of normality [94]. To take into account potential seasonal influences on rodent behavior, we initially included a seasonality variable in our models; defined as the difference in days between a baseline date (July 1st 2018) and the date of the trials. This seasonality variable had no significant effects on any of our behavioral responses, so we excluded it from all successive models to not exhaust too many degrees of freedom. Additionally, because species already considers differences in life history and morphological traits, such as size, we did not include body weight as a factor in our models.

To test responses to a novel environment (first trial only), for all the duration-based response variables, with the exception of *exploring* and *non-defensive* behaviors, we ran a GLM incorporating a negative binomial log-link function with a fixed offset value equal to 2100 (total amount of time in seconds in a trial). In these models, the predictors were fixed factors species and sex, as well as their interaction. The response variables *exploring* and *non-defensive* were analyzed using a GLM fit with a gamma log-link function; with species and sex set as the fixed factors.

We measured rodent responses to leopard cat odor using a GEE (with the exception of *contact*) with test animal ID as the subject variable and trial as the within-subject variable. GEEs with negative binomial log-link functions were performed for each duration-based response incorporating an offset equal to 2100 and setting trial (first and second), treatment (leopard cat odor or control), and species as the fixed factors. The variable trial was included in our models as a factor because in addition to testing the effects of a predator odor, we sought to assess any differences in behaviors between the first and second trials. We included two-way interactions between our fixed factors in our models, but not a three-way interaction. We excluded a three-way interaction because it did not coincide with our research objectives and to save degrees of freedom. The variable sex was excluded from these models since animal ID was already included, which factored in sex as well as the other unique characteristics of an individual animal. Additionally, testing the effect of sex was not an objective of ours for the second trial. For the response *contact* we ran a GLM with a negative binomial log-link function and included treatment and species as fixed factors, since this variable was only measured during the second trial. Post hoc analyses were performed for all models to test for differences between subgroups (e.g. species groups, trials of a species) of our fixed factors using estimated marginal means with a pairwise contrast incorporating a least significant difference.

In order to assess the potential importance of within-individual consistency for our measured behaviors [14, 15, 17, 82], we performed two comparisons of linear mixed models (LMMs) with the restricted maximum likelihood (REML) method; each comprising two models. In the first comparison, one model only included intercept, and the second model included intercept and rodent identity (ID) set as a random effect. This model provided a baseline for amount of variance explained by rodent ID (Table S5). In the second comparison, both models included species, trial and treatment as fixed effects, and only one model included ID as a random effect. For each comparison, the two models (with or without ID as a random effect) were tested for significance of between-individual variance of a behavior by calculating the log-likelihood ratio [15, 17, 82]. We approximated the *p*-value of the log-likelihood ratio test (LRT) following Martin & Reale [15]. For each behavior, repeatability was estimated as *R* = V_i_/(V_i_ + V_r_); where V_i_ is the variance of the random effect (rodent identity) and V_r_ is the residual variance [20, 82]. Please refer to the Supplementary material for the results (Table S5 and S6).

For all of our statistical analyses significance was considered at α = 0.05. All statistical analyses were performed with SPSS *v.25.0* (IBM, Armonk, USA).

## Supplementary information

**Additional file 1: Table S1**. Mean and standard error in duration (sec.), amount (%) and frequency of response behaviors of species and sexes for Trial 1. **Table S2**. Mean and standard error in duration (sec.), amount (%) and frequency of response behaviors of species and treatment groups for Trial 2. **Table S3**. Spearman rank correlation matrix of all measured individual behaviors. Significant values are displayed in bold. **Table S4.** Behavioral responses of first trial only for the factor species, sex, and their interaction; and behavioral responses for the effects and interactions trial, treatment, and species. Significant values are displayed in bold. **Table S5**. Within-individual consistency in behaviors and significance of a random effect (ID) in linear mixed models of behavioral variables for individuals from all species (*n* = 68). Significant differences between models are based on log-likelihood ratio tests and displayed in bold. **Table S6**. Within-individual consistency in behaviors and significance of a random effect (ID) in linear mixed models of behavioral variables for individuals from all species (n = 68). Species, treatment and trial included as fixed effects in both models. Significant differences between models are based on log-likelihood ratio tests and displayed in bold. **Figure S1**. Mean duration (out of 2100 s) of behaviors A) Concealing and B) Latency to forage, number of occurrences of C) Foraging events, and D) Consumption ratio. Each response variable is compared between species and trial. Error bars represent standard error of the mean. Differences in letters above each species indicates significant differences based on post hoc analysis between 1st trial and 2nd trial for that species. **Figure S2**. Mean number of occurrences of A) Jumping, and B) Contact. Jumping is compared between species, treatment group and trial. Contact is compared between species and treatment group. 1st trial comprises both treatment groups. ‘2nd trial C’ refers to the control group during the second trial. ‘2nd trial LC’ refers to the leopard cat odor group during the second trial. Error bars represent standard error of the mean. Differences in letters above each species indicates significant differences based on post hoc analysis between subgroups: 1st trial, 2nd trial C, 2nd trial LC.

## Data Availability

The datasets used and analyzed during the current study are available from the corresponding author on request.

## Abbreviations

GLM: Generalized linear model
GEE: Generalized estimating equation
LMM: Linear mixed model
LRT: Log-likelihood ratio test
POL: Pace-of-life
REML: Restricted maximum likelihood
