# Supplementary material for: Bigger doesn’t mean bolder: behavioral variation of four wild rodent species to novelty and predation risk following a fast-slow continuum
Source: Front Zool. 2020 Sep 21;17:27. doi: 10.1186/s12983-020-00376-8 (PMC7507744; doi:10.1186/s12983-020-00376-8)
Supplement: Supplementary file 1 — Additional file 1: Table S1. Mean and standard error in duration (sec.), amount (%) and frequency of response behaviors of species and sexes for Trial 1. Table S2. Mean and standard error in duration (sec.), amount (%) and frequency of response behaviors of species and treatment groups for Trial 2. Table S3. Spearman rank correlation matrix of all measured individual behaviors. Significant values are displayed in bold. Table S4. Behavioral responses of first trial only for the factor species, sex, and their interaction; and behavioral responses for the effects and interactions trial, treatment, and species. Significant values are displayed in bold. Table S5. Within-individual consistency in behaviors and significance of a random effect (ID) in linear mixed models of behavioral variables for individuals from all species (n = 68). Significant differences between models are based on log-likelihood ratio tests and displayed in bold. Table S6. Within-individual consistency in behaviors and significance of a random effect (ID) in linear mixed models of behavioral variables for individuals from all species (n = 68). Species, treatment and trial included as fixed effects in both models. Significant differences between models are based on log-likelihood ratio tests and displayed in bold. Figure S1. Mean duration (out of 2100 s) of behaviors A) Concealing and B) Latency to forage, number of occurrences of C) Foraging events, and D) Consumption ratio. Each response variable is compared between species and trial. Error bars represent standard error of the mean. Differences in letters above each species indicates significant differences based on post hoc analysis between 1st trial and 2nd trial for that species. Figure S2. Mean number of occurrences of A) Jumping, and B) Contact. Jumping is compared between species, treatment group and trial. Contact is compared between species and treatment group. 1st trial comprises both treatment groups. ‘2nd trial C’ refers to the control gr [file 12983_2020_376_MOESM1_ESM.docx]

**Table S1**. Mean and standard error in duration (sec.), amount (%) and frequency of response behaviors of species and sexes for Trial 1

|  |  |  | **^a^Expl.** | **^a^For.** | **^a^Mot.** | **^a^Conc.** | **^a^Groom.** | **^a^Def.** | **^a^Non-def.** | **^b^Cons.R.** | **^c^For. Ev.** | **^a^Lat. For.** | **^c^Jump.** |
| --- | --- | --- | --- | --- | --- | --- | --- | --- | --- | --- | --- | --- | --- |
| ***M. caroli*** | Male | Mean | 1428.20 | 388.60 | 208.40 | 53.40 | 21.40 | 261.80 | 1816.80 | 0.32 | 0.40 | 1327.60 | 41.20 |
|  |  | S.E. | 350.51 | 384.86 | 118.64 | 36.82 | 7.03 | 149.81 | 147.95 | 0.32 | 0.24 | 473.00 | 25.13 |
|  | Female | Mean | 1704.00 | 168.00 | 24.50 | 154.38 | 49.13 | 178.88 | 1872.00 | 0.31 | 1.75 | 1567.13 | 74.88 |
|  |  | S.E. | 169.44 | 139.70 | 11.71 | 126.70 | 14.08 | 125.39 | 125.13 | 0.21 | 1.08 | 275.78 | 22.83 |
|  | Total | Mean | 1597.92 | 252.85 | 95.23 | 115.54 | 38.46 | 210.77 | 1850.77 | 0.31 | 1.23 | 1475.00 | 61.92 |
|  |  | S.E. | 166.01 | 164.18 | 50.21 | 78.34 | 9.63 | 93.04 | 92.13 | 0.17 | 0.68 | 238.99 | 17.04 |
| ***A. agrarius*** | Male | Mean | 625.43 | 1271.14 | 29.86 | 103.86 | 69.71 | 133.71 | 1896.57 | 2.90 | 9.14 | 158.29 | 0.14 |
|  |  | S.E. | 63.89 | 60.07 | 13.38 | 75.90 | 29.93 | 73.95 | 97.81 | 0.53 | 1.39 | 31.57 | 0.14 |
|  | Female | Mean | 531.11 | 1187.22 | 83.67 | 261.22 | 36.78 | 344.89 | 1718.33 | 2.33 | 5.00 | 485.56 | 1.00 |
|  |  | S.E. | 79.95 | 144.32 | 67.89 | 172.29 | 14.92 | 175.79 | 168.46 | 0.54 | 0.76 | 139.47 | 0.88 |
|  | Total | Mean | 572.38 | 1223.94 | 60.13 | 192.38 | 51.19 | 252.50 | 1796.31 | 2.58 | 6.81 | 342.38 | 0.63 |
|  |  | S.E. | 52.71 | 83.64 | 38.23 | 101.59 | 15.53 | 104.69 | 103.48 | 0.38 | 0.89 | 88.13 | 0.50 |
| ***R. exulans*** | Male | Mean | 767.00 | 591.20 | 45.53 | 649.47 | 46.80 | 695.00 | 1358.20 | 1.37 | 6.40 | 867.53 | 16.80 |
|  |  | S.E. | 96.64 | 142.29 | 13.66 | 169.18 | 22.83 | 166.90 | 173.48 | 0.33 | 1.43 | 239.99 | 10.07 |
|  | Female | Mean | 1006.14 | 339.43 | 227.43 | 438.86 | 88.14 | 666.29 | 1345.57 | 0.86 | 2.86 | 1244.43 | 29.00 |
|  |  | S.E. | 179.93 | 172.81 | 107.82 | 176.53 | 39.54 | 178.03 | 190.36 | 0.48 | 1.61 | 329.22 | 19.63 |
|  | Total | Mean | 843.09 | 511.09 | 103.41 | 582.45 | 59.95 | 685.86 | 1354.18 | 1.21 | 5.27 | 987.45 | 20.68 |
|  |  | S.E. | 88.20 | 112.13 | 38.52 | 127.67 | 19.92 | 124.70 | 130.29 | 0.27 | 1.14 | 193.65 | 9.09 |
| ***R. losea*** | Male | Mean | 680.00 | 891.30 | 388.40 | 5.90 | 134.40 | 394.30 | 1571.30 | 1.37 | 6.00 | 523.20 | 15.60 |
|  |  | S.E. | 82.96 | 131.35 | 107.22 | 5.37 | 53.05 | 106.01 | 114.59 | 0.26 | 0.91 | 212.89 | 6.25 |
|  | Female | Mean | 726.00 | 24.29 | 636.43 | 311.86 | 401.43 | 948.29 | 750.29 | 0.05 | 0.14 | 2075.71 | 12.86 |
|  |  | S.E. | 94.35 | 24.29 | 143.88 | 203.13 | 66.48 | 122.05 | 87.40 | 0.05 | 0.14 | 24.29 | 11.37 |
|  | Total | Mean | 698.94 | 534.29 | 490.53 | 131.88 | 244.35 | 622.41 | 1233.24 | 0.82 | 3.59 | 1162.47 | 14.47 |
|  |  | S.E. | 60.70 | 131.07 | 89.06 | 88.31 | 51.89 | 103.27 | 125.41 | 0.22 | 0.89 | 227.10 | 5.75 |
| **Total** | Male | Mean | 806.05 | 773.57 | 157.24 | 291.76 | 71.38 | 449.00 | 1579.62 | 1.52 | 6.00 | 702.46 | 16.62 |
|  |  | S.E. | 75.31 | 95.03 | 40.74 | 84.67 | 18.64 | 83.77 | 86.91 | 0.22 | 0.78 | 138.22 | 5.64 |
|  | Female | Mean | 985.06 | 470.16 | 225.68 | 285.19 | 133.90 | 510.87 | 1455.23 | 0.96 | 2.58 | 1295.10 | 29.06 |
|  |  | S.E. | 105.06 | 107.47 | 60.32 | 82.89 | 31.73 | 91.00 | 105.43 | 0.25 | 0.58 | 150.31 | 9.08 |
|  | Total | Mean | 887.66 | 635.25 | 188.44 | 288.76 | 99.88 | 477.21 | 1522.91 | 1.26 | 4.44 | 972.63 | 22.29 |
|  |  | S.E. | 63.48 | 73.07 | 35.29 | 59.15 | 17.93 | 61.29 | 67.34 | 0.17 | 0.54 | 107.23 | 5.17 |

**Notes**: Expl. = exploring, For. = foraging, Mot. = motionless, Conc. = concealing, Groom. = grooming, Def. = defensive, Non-def. = non-defensive, Cons.R. = consumption ratio, For. Ev. = foraging events, Lat. For. = latency to forage, Jump. = jumping. ^a^ Duration-based response (out of 2100 sec.); ^b^ amount (%); ^c^ number of occurrences. S.E. = standard error of mean.

**Table S2**. Mean and standard error in duration (sec.), amount (%) and frequency of response behaviors of species and treatment groups for Trial 2

|  |  |  | **^a^Expl.** | **^a^For.** | **^a^Mot.** | **^a^Conc.** | **^a^Groom.** | **^a^Def.** | **^a^Non-def.** | **^b^Cons.R.** | **^c^For. Ev.** | **^a^Lat. For.** | **^c^Jump.** | **^c^Cont.** |
| --- | --- | --- | --- | --- | --- | --- | --- | --- | --- | --- | --- | --- | --- | --- |
| ***M. caroli*** | Control | Mean | 1350.57 | 520.14 | 3.57 | 199.14 | 26.57 | 202.71 | 1870.71 | 0.77 | 2.86 | 1360.43 | 82.57 | 8.00 |
|  |  | S.E. | 219.79 | 243.17 | 2.83 | 72.48 | 11.94 | 71.41 | 71.43 | 0.41 | 1.53 | 333.65 | 29.98 | 1.73 |
|  | LC odor | Mean | 1645.67 | 258.67 | 7.00 | 152.67 | 36.00 | 159.67 | 1904.33 | 1.33 | 4.33 | 1425.50 | 47.00 | 7.50 |
|  |  | S.E. | 187.39 | 192.09 | 2.24 | 119.74 | 14.37 | 120.45 | 114.52 | 0.94 | 2.62 | 422.94 | 18.84 | 0.85 |
|  | Total | Mean | 1486.77 | 399.46 | 5.15 | 177.69 | 30.92 | 182.85 | 1886.23 | 1.03 | 3.54 | 1390.46 | 66.15 | 7.77 |
|  |  | S.E. | 146.84 | 156.31 | 1.83 | 64.93 | 8.94 | 64.82 | 62.60 | 0.47 | 1.41 | 253.89 | 18.34 | 0.98 |
| ***A. agrarius*** | Control | Mean | 175.50 | 1804.38 | 21.75 | 31.50 | 66.88 | 53.25 | 1979.88 | 4.50 | 3.25 | 66.13 | 0.00 | 2.00 |
|  |  | S.E. | 58.39 | 89.90 | 8.18 | 15.38 | 37.61 | 13.73 | 36.72 | 0.34 | 0.77 | 15.11 | 0.00 | 0.68 |
|  | LC odor | Mean | 182.25 | 1436.63 | 61.00 | 365.50 | 54.63 | 426.50 | 1618.88 | 4.04 | 3.38 | 152.38 | 0.00 | 2.88 |
|  |  | S.E. | 43.70 | 178.71 | 47.39 | 176.56 | 45.71 | 164.47 | 167.84 | 0.44 | 0.42 | 38.73 | 0.00 | 0.30 |
|  | Total | Mean | 178.88 | 1620.50 | 41.38 | 198.50 | 60.75 | 239.88 | 1799.38 | 4.27 | 3.31 | 109.25 | 0.00 | 2.44 |
|  |  | S.E. | 35.24 | 107.67 | 23.77 | 95.86 | 28.64 | 93.15 | 95.18 | 0.28 | 0.43 | 22.96 | 0.00 | 0.38 |
| ***R. exulans*** | Control | Mean | 789.36 | 846.27 | 53.00 | 390.82 | 20.55 | 443.82 | 1635.64 | 1.46 | 5.00 | 824.91 | 21.91 | 5.00 |
|  |  | S.E. | 194.32 | 246.31 | 27.67 | 146.33 | 7.47 | 152.59 | 151.53 | 0.39 | 1.73 | 305.52 | 10.50 | 1.10 |
|  | LC odor | Mean | 630.36 | 1078.00 | 8.00 | 376.82 | 6.82 | 384.82 | 1708.36 | 2.20 | 5.09 | 505.73 | 33.55 | 4.18 |
|  |  | S.E. | 198.43 | 240.31 | 3.52 | 154.78 | 3.02 | 155.27 | 154.64 | 0.52 | 1.42 | 244.46 | 25.18 | 1.18 |
|  | Total | Mean | 709.86 | 962.14 | 30.50 | 383.82 | 13.68 | 414.32 | 1672.00 | 1.83 | 5.05 | 665.32 | 27.73 | 4.59 |
|  |  | S.E. | 136.63 | 169.81 | 14.47 | 103.94 | 4.21 | 106.42 | 105.94 | 0.33 | 1.09 | 194.08 | 13.37 | 0.79 |
| ***R. losea*** | Control | Mean | 897.13 | 389.00 | 447.75 | 261.13 | 105.00 | 708.88 | 1286.13 | 1.25 | 4.50 | 940.75 | 49.63 | 3.75 |
|  |  | S.E. | 165.37 | 160.79 | 229.64 | 197.24 | 20.35 | 249.58 | 247.65 | 0.50 | 1.89 | 357.62 | 10.52 | 0.84 |
|  | LC odor | Mean | 752.00 | 531.11 | 282.89 | 456.44 | 77.56 | 739.33 | 1283.11 | 1.03 | 4.67 | 629.44 | 2.56 | 4.11 |
|  |  | S.E. | 190.22 | 178.76 | 117.25 | 301.47 | 29.07 | 269.04 | 256.03 | 0.34 | 1.41 | 279.09 | 1.53 | 1.07 |
|  | Total | Mean | 820.29 | 464.24 | 360.47 | 364.53 | 90.47 | 725.00 | 1284.53 | 1.14 | 4.59 | 775.94 | 24.71 | 3.94 |
|  |  | S.E. | 124.64 | 118.73 | 122.15 | 180.73 | 17.91 | 178.88 | 173.14 | 0.29 | 1.12 | 220.13 | 7.61 | 0.67 |
| **Total** | Control | Mean | 785.82 | 896.97 | 128.35 | 236.29 | 52.56 | 364.65 | 1682.79 | 1.99 | 4.03 | 783.88 | 35.76 | 4.62 |
|  |  | S.E. | 108.53 | 135.93 | 60.60 | 69.25 | 11.72 | 85.75 | 86.97 | 0.32 | 0.78 | 160.16 | 8.72 | 0.65 |
|  | LC odor | Mean | 736.29 | 873.03 | 93.06 | 355.68 | 41.94 | 448.74 | 1609.32 | 2.17 | 4.44 | 617.65 | 19.82 | 4.44 |
|  |  | S.E. | 118.07 | 126.20 | 37.45 | 102.11 | 13.73 | 99.57 | 98.61 | 0.33 | 0.73 | 144.47 | 9.10 | 0.55 |
|  | Total | Mean | 761.06 | 885.00 | 110.71 | 295.99 | 47.25 | 406.69 | 1646.06 | 2.08 | 4.24 | 700.76 | 27.79 | 4.53 |
|  |  | S.E. | 79.64 | 92.06 | 35.42 | 61.66 | 8.98 | 65.41 | 65.40 | 0.23 | 0.53 | 107.52 | 6.33 | 0.42 |

**Notes**: LC odor = leopard cat odor group; Expl. = exploring, For. = foraging, Mot. = motionless, Conc. = concealing, Groom. = grooming, Def. = defensive, Non-def. = non-defensive, Cons.R. = consumption ratio, For. Ev. = foraging events, Lat. For. = latency to forage, Jump. = jumping, Cont. = contact. ^a^ Duration-based response (out of 2100 sec.); ^b^ amount (%); ^c^ number of occurrences. S.E. = standard error of mean.

| **Table S3**. Spearman rank correlation matrix of all measured individual behaviors. Significant values are displayed in bold. | | | | | | | | | |
| --- | --- | --- | --- | --- | --- | --- | --- | --- | --- |
|  | Expl. | For. | Mot. | Conc. | Groom. | Cons. R. | For. Ev. | Lat. For. | Jump. |
| Expl. | 1 | -0.621 | 0.088 | -0.065 | 0.284 | -0.504 | -0.148 | 0.460 | 0.765 |
|  |  | **<0.001** | 0.310 | 0.450 | **<0.001** | **<0.001** | 0.085 | **<0.001** | **<0.001** |
| For. | -0.621 | 1 | -0.235 | -0.319 | -0.335 | 0.875 | 0.613 | -0.838 | -0.580 |
|  | **<0.001** |  | **<0.01** | **<0.001** | **<0.001** | **<0.001** | **<0.001** | **<0.001** | **<0.001** |
| Mot. | 0.088 | -0.235 | 1 | -0.384 | 0.478 | -0.242 | -0.129 | 0.209 | 0.038 |
|  | 0.310 | **<0.01** |  | **<0.001** | **<0.001** | **<0.01** | 0.133 | **<0.05** | 0.660 |
| Conc. | -0.065 | -0.319 | -0.384 | 1 | -0.208 | -0.255 | -0.199 | 0.245 | 0.103 |
|  | 0.450 | **<0.001** | **<0.001** |  | **<0.05** | **<0.005** | **<0.05** | **<0.005** | 0.231 |
| Groom. | 0.284 | -0.335 | 0.478 | -0.208 | 1 | -0.264 | -0.184 | 0.311 | 0.268 |
|  | **<0.001** | **<0.001** | **<0.001** | **<0.05** |  | **<0.005** | **<0.05** | **<0.001** | **<0.005** |
| Cons. R. | -0.504 | 0.875 | -0.242 | -0.255 | -0.264 | 1 | 0.717 | -0.842 | -0.476 |
|  | **<0.001** | **<0.001** | **<0.01** | **<0.01** | **<0.005** |  | **<0.001** | **<0.001** | **<0.001** |
| For. Ev. | -0.148 | 0.613 | -0.129 | -0.199 | -0.184 | 0.717 | 1 | -0.729 | -0.270 |
|  | 0.085 | **<0.001** | 0.133 | **<0.05** | <0.05 | **<0.001** |  | **<0.001** | **<0.005** |
| Lat. For. | 0.460 | -0.838 | 0.209 | 0.245 | 0.311 | -0.842 | -0.729 | 1 | 0.441 |
|  | **<0.001** | **<0.001** | **<0.05** | **<0.005** | **<0.001** | **<0.001** | **<0.001** |  | **<0.001** |
| Jump. | 0.765 | -0.580 | 0.038 | 0.103 | 0.268 | -0.476 | -0.270 | 0.441 | 1 |
|  | **<0.001** | **<0.001** | 0.660 | 0.231 | **<0.005** | **<0.001** | **<0.005** | **<0.001** |  |

**Notes**: Expl. = exploring, For. = foraging, Mot. = motionless, Conc. = concealing, Groom. = grooming, Cons. R. = consumption ratio, For. Ev. = foraging events, Lat. For. = latency to forage, Jump. = jumping.

| **Table S4.** Behavioral responses of first trial only for the factor species, sex, and their interaction; and behavioral responses for the effects and interactions trial, treatment, and species. Significant values are displayed in bold. | | | | | | | | |
| --- | --- | --- | --- | --- | --- | --- | --- | --- |
| **Response** | **First Trial^1^** | | | | **Both Trials^2^** | | | |
|  | **Factor** | **Wald χ^2^** | ***df*** | ***P*** | **Factor** | **Wald χ^2^** | ***df*** | ***P*** |
| *Consumption ratio*^a^ | Species | 21.20 | 3 | **<0.001** | Trial | 27.32 | 1 | **<0.001** |
|  | Sex | 4.19 | 1 | **<0.05** | Treatment | 0.05 | 1 | 0.818 |
|  | Species × Sex | 5.88 | 3 | 0.118 | Species | 90.46 | 3 | **<0.001** |
|  |  |  |  |  | Trial × Treatment | 0.35 | 1 | 0.553 |
|  |  |  |  |  | Trial × Species | 8.38 | 3 | **<0.05** |
|  |  |  |  |  | Treatment × Species | 6.92 | 3 | 0.075 |
| *Foraging events*^b^ | Species | 21.24 | 3 | **<0.001** | Trial | 1.32 | 1 | 0.251 |
|  | Sex | 4.96 | 1 | **<0.05** | Treatment | 0.70 | 1 | 0.403 |
|  | Species × Sex | 12.68 | 3 | **<0.01** | Species | 4.66 | 3 | 0.199 |
|  |  |  |  |  | Trial × Treatment | 0.33 | 1 | 0.563 |
|  |  |  |  |  | Trial × Species | 26.65 | 3 | **<0.001** |
|  |  |  |  |  | Treatment × Species | 0.37 | 3 | 0.947 |
| *Latency to forage*^a^ | Species | 29.41 | 3 | **<0.001** | Trial | 26.17 | 1 | **<0.001** |
|  | Sex | 10.69 | 1 | **<0.005** | Treatment | 0.02 | 1 | 0.878 |
|  | Species × Sex | 4.73 | 3 | 0.193 | Species | 90.44 | 3 | **<0.001** |
|  |  |  |  |  | Trial × Treatment | 0.28 | 1 | 0.600 |
|  |  |  |  |  | Trial × Species | 14.89 | 3 | **<0.005** |
|  |  |  |  |  | Treatment × Species | 6.21 | 3 | 0.102 |
| *Jumping*^b^ | Species | 60.59 | 3 | **<0.001** | Trial | 0.07 | 1 | 0.789 |
|  | Sex | 3.86 | 1 | 0.052 | Treatment | 0.05 | 1 | 0.825 |
|  | Species × Sex | 3.32 | 3 | 0.345 | Species | 67.30 | 3 | **<0.001** |
|  |  |  |  |  | Trial × Treatment | 0.30 | 1 | 0.584 |
|  |  |  |  |  | Trial × Species | 0.33 | 3 | 0.847 |
|  |  |  |  |  | Treatment × Species | 13.19 | 3 | **<0.005** |

**Notes**: ^1 a^ performed with GLM with gamma log-link function; ^1 b^ performed with GLM with negative binomial log-link function; ^2 a^ performed with GEE with gamma log-link function; ^2 b^ performed with GEE with negative binomial log-link function.

| **Table S5**. Within-individual consistency in behaviors and significance of a random effect (ID) in linear mixed models of behavioral variables for individuals from all species (n = 68). Significant differences between models are based on log-likelihood ratio tests and displayed in bold. | | | | | | | | | | |
| --- | --- | --- | --- | --- | --- | --- | --- | --- | --- | --- |
| Behavior | ID | Model | *K* | -2LL | Test | LRT | *df* | *P* | *R* | |
| *Defensive* |  | 1 | 1 | 2077.57 |  |  |  |  |  | |
|  | x | 2 | 2 | 2067.14 | 1 vs. 2 | 10.43 | 1 | **<0.005** | 0.378 | |
| *Non-defensive* |  | 1 | 1 | 2091.13 |  |  |  |  |  | |
|  | x | 2 | 2 | 2077.96 | 1 vs. 2 | 13.17 | 1 | **<0.001** | 0.421 | |
| *Exploring* |  | 1 | 1 | 2112.97 |  |  |  |  |  | |
|  | x | 2 | 2 | 2095.87 | 1 vs. 2 | 17.10 | 1 | **<0.001** | 0.473 | |
| *Foraging* |  | 1 | 1 | 2154.56 |  |  |  |  |  | |
|  | x | 2 | 2 | 2116.74 | 1 vs. 2 | 37.82 | 1 | **<0.001** | 0.654 | |
| *Motionless* |  | 1 | 1 | 1921.72 |  |  |  |  |  | |
|  | x | 2 | 2 | 1896.59 | 1 vs. 2 | 25.13 | 1 | **<0.001** | 0.557 | |
| *Concealing* |  | 1 | 1 | 2064.02 |  |  |  |  |  | |
|  | x | 2 | 2 | 2057.08 | 1 vs. 2 | 6.22 | 1 | **<0.05** | 0.312 | |
| *Grooming* |  | 1 | 1 | 1679.45 |  |  |  |  |  | |
|  | x | 2 | 2 | 1675.97 | 1 vs. 2 | 3.48 | 1 | 0.062 | 0.224 | |
| *Consumption ratio* |  | 1 | 1 | 528.83 |  |  |  |  | |  |
|  | x | 2 | 2 | 505.12 | 1 vs. 2 | 23.71 | 1 | **<0.001** | | 0.544 |
| *Foraging events* |  | 1 | 1 | 788.00 |  |  |  |  | |  |
|  | x | 2 | 2 | 777.99 | 1 vs. 2 | 10.01 | 1 | **<0.005** | | 0.371 |
| *Latency to forage* |  | 1 | 1 | 2222.45 |  |  |  |  | |  |
|  | x | 2 | 2 | 2171.70 | 1 vs. 2 | 50.75 | 1 | **<0.001** | | 0.726 |
| *Jumping* |  | 1 | 1 | 1430.65 |  |  |  |  |  | |
|  | x | 2 | 2 | 1393.55 | 1 vs. 2 | 37.10 | 1 | **<0.001** | 0.649 | |

Notes: ‘x’ indicates a random effect included in a model. ID = individual identity, *K* = number of parameters, -2LL = -2 log-likelihood, LRT = log-likelihood ratio test. *R* refers to repeatability, estimated as *R* = V_i_/(V_i_ + V_r_), where V_i_ is variance of the random effect (ID) and V_r_ is residual variance of the model.

| **Table S6**. Within-individual consistency in behaviors and significance of a random effect (ID) in linear mixed models of behavioral variables for individuals from all species (n = 68). Species, treatment and trial included as fixed effects in both models. Significant differences between models are based on log-likelihood ratio tests and displayed in bold. | | | | | | | | | |
| --- | --- | --- | --- | --- | --- | --- | --- | --- | --- |
| Behavior | ID | Model | *K* | -2LL | Test | LRT | *df* | *P* | *R* |
| *Defensive* |  | 1 | 3 | 2001.18 |  |  |  |  |  |
|  | x | 2 | 4 | 1994.93 | 1 vs. 2 | 6.25 | 1 | **<0.05** | 0.302 |
| *Non-defensive* |  | 1 | 3 | 2006.37 |  |  |  |  |  |
|  | x | 2 | 4 | 1998.84 | 1 vs. 2 | 7.53 | 1 | **<0.01** | 0.330 |
| *Exploring* |  | 1 | 3 | 1981.93 |  |  |  |  |  |
|  | x | 2 | 4 | 1980.85 | 1 vs. 2 | 1.08 | 1 | 0.299 | 0.128 |
| *Foraging* |  | 1 | 3 | 2038.12 |  |  |  |  |  |
|  | x | 2 | 4 | 2011.60 | 1 vs. 2 | 25.62 | 1 | **<0.001** | 0.576 |
| *Motionless* |  | 1 | 3 | 1820.40 |  |  |  |  |  |
|  | x | 2 | 4 | 1808.16 | 1 vs. 2 | 12.24 | 1 | **<0.001** | 0.413 |
| *Concealing* |  | 1 | 3 | 1996.55 |  |  |  |  |  |
|  | x | 2 | 4 | 1991.77 | 1 vs. 2 | 4.78 | 1 | **<0.05** | 0.266 |
| *Grooming* |  | 1 | 3 | 1599.23 |  |  |  |  |  |
|  | x | 2 | 4 | 1598.53 | 1 vs. 2 | 0.7 | 1 | 0.403 | 0.104 |
| *Consumption ratio* |  | 1 | 3 | 457.45 |  |  |  |  |  |
|  | x | 2 | 4 | 444.58 | 1 vs. 2 | 12.87 | 1 | **<0.001** | 0.422 |
| *Foraging events* |  | 1 | 3 | 771.79 |  |  |  |  |  |
|  | x | 2 | 4 | 763.18 | 1 vs. 2 | 8.61 | 1 | **<0.005** | 0.351 |
| *Latency to forage* |  | 1 | 3 | 2127.39 |  |  |  |  |  |
|  | x | 2 | 4 | 2129.42 | 1 vs. 2 | 47.35 | 1 | **<0.001** | 0.716 |
| *Jumping* |  | 1 | 3 | 1367.16 |  |  |  |  |  |
|  | x | 2 | 4 | 1340.60 | 1 vs. 2 | 26.56 | 1 | **<0.001** | 0.576 |

Notes: ‘x’ indicates a random effect included in a model. ID = individual identity, *K* = number of parameters, -2LL = -2 log-likelihood, LRT = log-likelihood ratio test. *R* refers to repeatability, estimated as *R* = V_i_/(V_i_ + V_r_), where V_i_ is variance of the random effect (ID) and V_r_ is residual variance of the model.


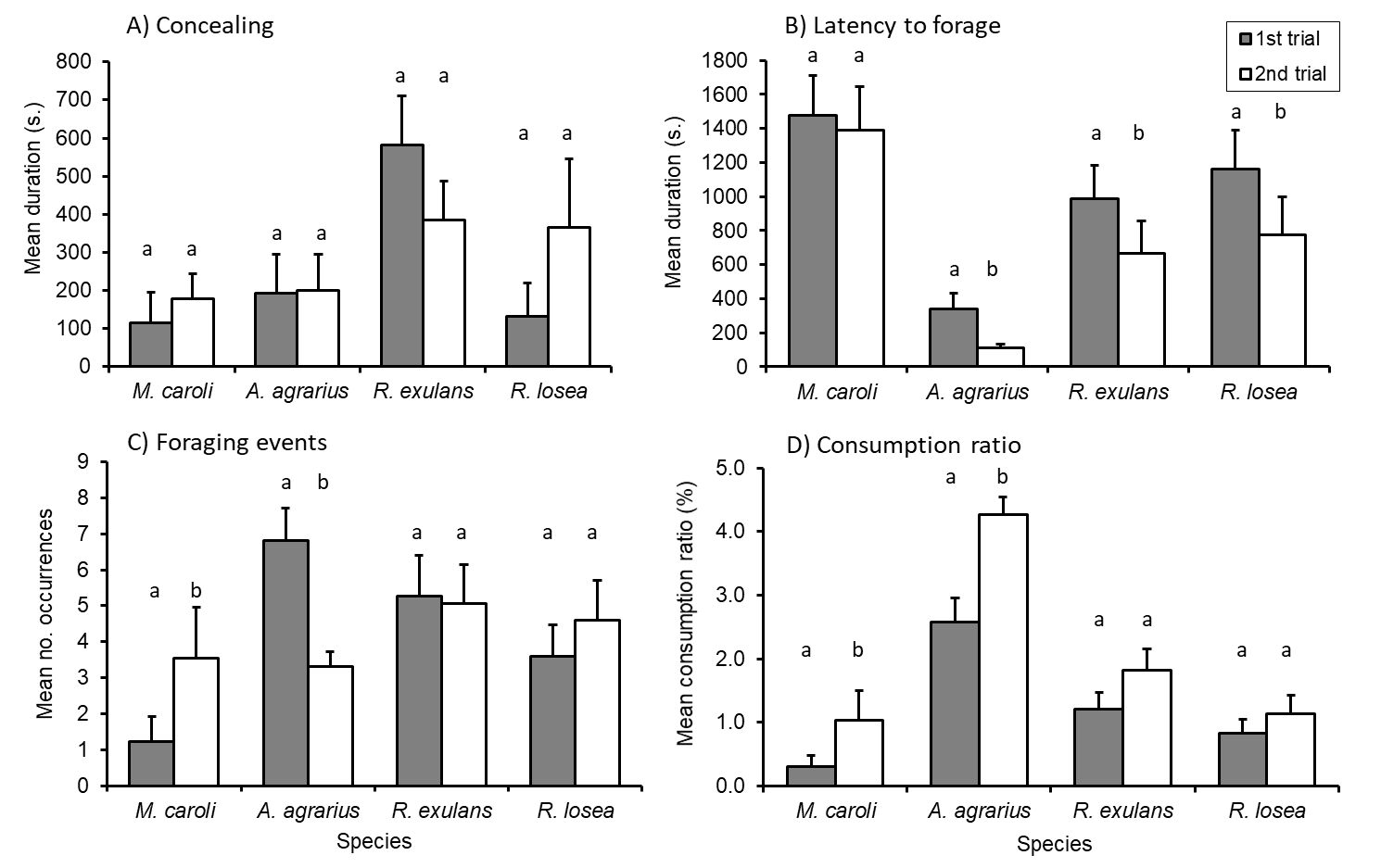


**Figure S1**. Mean duration (out of 2100 seconds) of behaviors A) Concealing and B) Latency to forage, number of occurrences of C) Foraging events, and D) Consumption ratio. Each response variable is compared between species and trial. Error bars represent standard error of the mean. Differences in letters above each species indicates significant differences based on post hoc analysis between 1st trial and 2nd trial for that species.


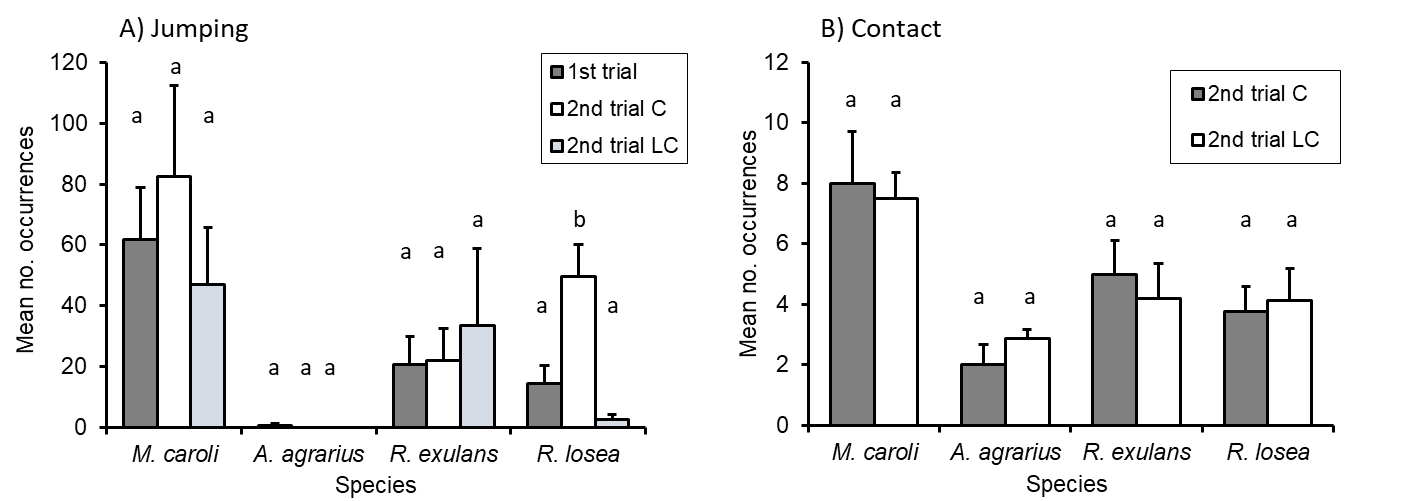


**Figure S2**. Mean number of occurrences of A) Jumping, and B) Contact. Jumping is compared between species, treatment group and trial. Contact is compared between species and treatment group. 1st trial comprises both treatment groups. ‘2nd trial C’ refers to the control group during the second trial. ‘2nd trial LC’ refers to the leopard cat odor group during the second trial. Error bars represent standard error of the mean. Differences in letters above each species indicates significant differences based on post hoc analysis between subgroups: 1st trial, 2nd trial C, 2nd trial LC.
